# Heterocyclic Analogues of Xanthone and Xanthione. 1*H*-Pyrano[2,3-*c*:6,5-*c*]dipyrazol-4(7*H*)-ones and Thiones: Synthesis and NMR Data

**DOI:** 10.3390/molecules15096106

**Published:** 2010-09-01

**Authors:** Barbara Datterl, Nicole Tröstner, Dorota Kucharski, Wolfgang Holzer

**Affiliations:** Department of Drug and Natural Product Synthesis, Faculty of Life Sciences, University of Vienna, Althanstrasse 14, A-1090 Vienna, Austria

**Keywords:** pyrazolones, 1*H*-pyrano[2,3-*c*:6,5-*c*]dipyrazol-4(7*H*)-ones, cyclization, Lawesson’s reagent, NMR spectroscopy

## Abstract

The synthesis of the title compounds is described. Reaction of 1-substituted 2-pyrazolin-5-ones with 5-chloro-1-phenyl-1*H*-pyrazole-4-carbonyl chloride or 5-chloro-3-methyl-1-phenyl-1*H*-pyrazole-4-carbonyl chloride, respectively, using calcium hydroxide in refluxing 1,4-dioxane gave the corresponding 4-heteroaroylpyrazol-5-ols, which were cyclized into 1*H*-pyrano[2,3-*c*:6,5-*c*]dipyrazol-4(7*H*)-ones by treatment with K_2_CO_3_/DMF. The latter were converted into the corresponding thiones upon reaction with Lawesson’s reagent. Detailed NMR spectroscopic investigations (^1^H, ^13^C, ^15^N) of the ring systems and their precursors are presented.

## 1. Introduction

**Figure 1:**
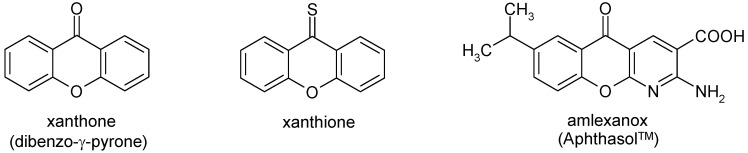
Xanthone, xanthione and its heterocyclic analogue amlexanox.

The xanthone system, shown in Figure 1, is the core of several biologically active compounds which play important roles in numerous biological processes [1,2]. Thus, for instance, xanthone derivatives with anti-cancer [3,4], cytotoxic [5,6], topoisomerase II inhibitory [5], monoamine oxidase inhibitory [6], antioxidant [6], and antimicrobial activity [7] have been described in the recent literature. In view of this fact synthetic derivatives of xanthones are attractive compounds for medicinal chemists. As a result analogues in which one or both benzene rings of the parent xanthone system had been replaced by heteroaromatic moieties were also studied. As a representative example of these compounds the anti-ulcer agent amlexanox (Figure 1) may be cited [8]. 

**Scheme 1:**
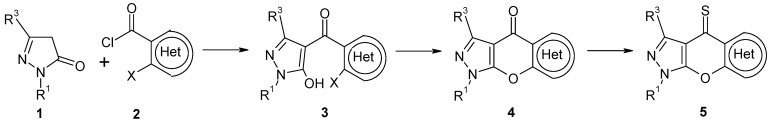
Synthesis of compounds **4** and **5**.

In the course of a program devoted to the synthesis of new heterocyclic scaffolds for bioactive compounds we recently presented the synthesis of various [5,6]pyrano[2,3-*c*]pyrazol-4(1*H*)-ones of type **4**, which can be considered as heterocyclic analogues of xanthone (Scheme 1) [9,10,11,12,13,14,15,16]. The synthesis of compounds **4** is based on the reaction of 2-pyrazolin-5-ones **1** with *o*-haloheteroarene-carbonyl chlorides **2** under the conditions described by Jensen for the C-4 acylation of pyrazolones (calcium hydroxide, dioxane, reflux) [17] and subsequent ring closure of the resulting 4-heteroaroyl-pyrazol-5-ols **3** (Scheme 1). Following this approach, we have obtained compounds of type **4** bearing – amongst others – a pyridine (all positional isomers) [9], quinoline [9], pyridazine [11], pyrimidine [11], pyrazine [15], thiophene (all positional isomers) [10,11], benzo[*b*]thiophene [10] and thieno[2,3-*b*]thiophene systems [11] as the variable heteroaromatic moiety (‘Het’) condensed to the central γ-pyranone ring. 

In continuation of these investigations we present here the synthesis and spectroscopic data of related congeners **4a**,**b** and **4d**-**g** containing a pyrazole system as the heteroaromatic moiety (‘Het’ = pyrazole) (Scheme 1), *i.e.* substituted 1*H*-pyrano[2,3-*c*:6,5-*c*]dipyrazol-4(7*H*)-ones. Moreover, the corresponding thiones **5a**,**b** and **5d**-**g** are described. Considering that thio analogues of flavones, isoflavones, xanthones (= xanthione, Figure 1) and related systems have received considerable attention due to the importance of such molecules in biology and photochemistry [18], the latter systems **5** are interesting compounds in their own right as well [19,20].

## 2. Results and Discussion

### 2.1. Chemistry

**Scheme 2:**
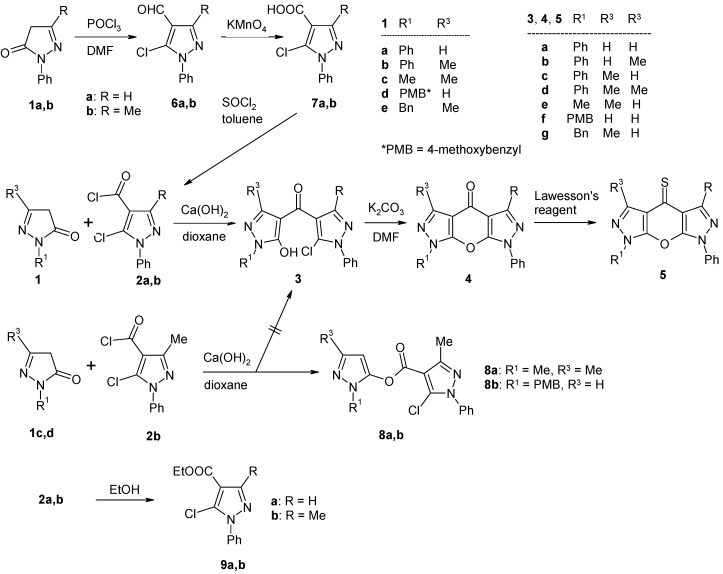
Synthesis of compounds **2**–**8**.

Synthesis of the target compounds **4** was accomplished via the sequence shown in Scheme 2. 2-Pyrazolin-5-ones **1** are either commercially available or can be easily prepared according to known methods [21]. Acid chlorides **2**, which can be considered as the key synthons in the approach presented, were prepared as follows: Vilsmaier-Haak formylation [22] of pyrazolones **1a** and **1b**, respectively, gave the 5-chloropyrazole-4-carbaldehydes **6**, which were oxidized to the corresponding carboxylic acids **7** by treatment with potassium permanganate [23]. Transformation of acids **7** into the appropriate acid chlorides **2** was accomplished with thionyl chloride in refluxing toluene [9,11,12]. Compounds **2** were always freshly prepared before reacting them with pyrazolones **1**; treatment of **7a**,**b** with dry ethanol led to esters **9a**,**b** [24] (Scheme 2).

**Scheme 3:**
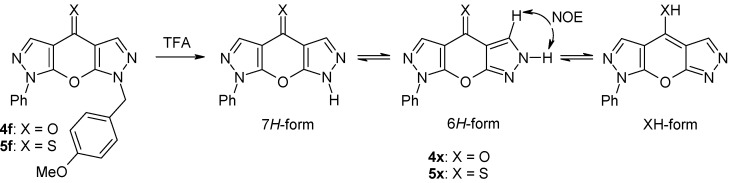
Synthesis of compounds **4x** and **5x** and their possible tautomeric forms.

Different pyrazolones **1a**-**e** were reacted with acid chlorides **2a**,**b** using calcium hydroxide in boiling dioxane [17] to afford the 4-pyrazoloylpyrazol-5-ols **3a-g** (Scheme 2). However, in two cases (the reactions of **1c** with **2b**, and **1d** with **2b**, respectively) the corresponding compounds of type **3** were not obtained, and instead, the isomeric esters **8a** and **8b** resulting from O-aroylation of **1c** and **1d** were isolated as the major products from the reaction mixtures. Their structures could be easily derived from the ^1^H-NMR spectra considering the characteristic singlet signal due to pyrazole H-4 at δ 6.04 (**8a**) and δ 6.30 ppm (**8b**). Attempts to convert compounds **8** into their corresponding 4-aroyl congeners **3** failed. Finally, cyclization of intermediates **3** under standard conditions (K_2_CO_3_ in DMF) [25] gave the target tricycles **4a**,**b **and **4d-g** in good yields. Treatment of the latter with Lawesson’s reagent [26,27,28] in refluxing toluene smoothly afforded the corresponding thiones **5a,b **and **5d-g**. It should be mentioned that compounds **4d** and **5d** have already been described by Sarenko and coworkers [29]. Finally, the N-7 unsubstituted compounds **4x** and **5x** were prepared by treatment of the corresponding N7-PMB-protected congeners **4f** and **5f**, respectively, with trifluoroacetic acid at 70 ºC [9,12] (Scheme 3).

### 2.2. NMR Spectroscopic Investigations

The NMR data of compounds **2**, **3**, **6**-**8** are given in the Experimental, whereas those of title compounds **4** and **5** are collected in Table 2, Table 3, Table 4 and Table 5 Unequivocal assignment of signals was carried out by the combined application of standard NMR spectroscopic techniques such as ^1^H-coupled ^13^C-NMR spectra, APT, HMQC, gs-HSQC, gs-HMBC, COSY, TOCSY, NOESY and NOE-difference spectroscopy [30]. Moreover, in a few cases experiments with selective excitation (DANTE) of certain ^1^H-resonances were performed, such as long-range INEPT [31] and 2D(δ,*J*) long-range INEPT [32], the latter experiments were indispensable for the unambiguous mapping of long-range ^13^C,^1^H coupling constants. Reliable and unambiguously assigned chemical shift data such as those presented here can be considered as important reference material for NMR prediction programs, such as CSEARCH [33]/NMRPREDICT [34] and ACD/C + H predictor [35] – programs which have become very popular in the last few years, particularly for predicting ^13^C-NMR chemical shifts.

**Figure 2:**
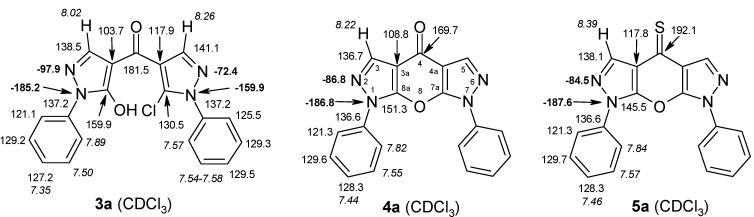
^1^H- (*in italics*), ^13^C- and ^15^N-NMR (in **bold**) chemical shifts in **3a**, **4a** and **5a** (δ, ppm, in CDCl_3_).

4-Aroylpyrazol-5-ols **3** in each case contain two different pyrazole units which exhibit characteristic differences regarding their chemical shift data. The 5-OH group in the hydroxypyrazole moiety leads to a strong polarization of the C4−C5 bond resulting in small chemical shifts for pyrazole C-4 (103−106 ppm) and large ones for pyrazole C-5 (159−161 ppm). These differences are significantly smaller in the 5-chloropyrazole unit with δ pyrazole C-4 having 117−119 ppm and pyrazole C-5 127−131 ppm. In congeners carrying phenyl substituents at both pyrazole N-1’s (compounds **3a**-**d**) an explicit difference regarding the resonances due to Ph C-2,6 is quite obvious (δ ~121 ppm in the 1-Ph-pyrazol-5-ol unit, δ ~125.5 ppm in the 1-Ph-5-Cl-pyrazole unit). Moreover, also the ^15^N-NMR chemical shifts in the mentioned pyrazole moieties differ markedly, both nitrogen atoms of the hydroxypyrazole system (for instance **3a**: N-1 −185.2 ppm, N-2 −97.9 ppm) show distinctly smaller chemical shifts than the corresponding ones in the chloropyrazole system (N-1 −159.9 ppm, N-2 −72.4 ppm). The C=O resonance in compounds **3** is located in the range from 181−184 ppm. In Figure 2, ^1^H, ^13^C and ^15^N-NMR chemical shift data are presented for **3a** which can be considered as a typical example.

In Figure 2, the chemical shift data for the corresponding tricycles **4a** and **5a** are also depicted, which − exemplarily – permit one to follow the changes when switching from the central pyran-4-one (**4a**) to a pyran-4-thione (**5a**) system. The transformation **4a** → **5a** leaves the ^1^H- and ^13^ C-NMR chemical shifts due to the phenyl ring nearly unchanged; δ (N-1), δ (N-2) and δ (C-3) are also only slightly affected. However, in **4a** a pronounced ‘push-pull situation’ is on hand which leads to a strong polarization of the pyrane C=C bond resulting in a large chemical shift for C7a/C8a (δ 151.3 ppm) and a small for C3a/C4a (δ 108.8 ppm). In **5a** this effect is much less pronounced leading to an upfield shift for C7a/C8a (δ 145.5 ppm) and a marked downfield shift for C3a/C4a (δ 117.8 ppm) compared to the appropriate shifts in **4a**. Expectedly, C-4 suffers a distinct downfield shift (169.7 ppm → 192.1 ppm) when switching from **4a** to **5a**, the difference of 22.4 ppm is comparable with corresponding values found in related systems [36]. 

Whereas assignment of signals in most cases is easy, the discrimination of signals due to N1-phenyl and N7-phenyl in ‘asymmetric’ compounds **4b** and **5b** is not trivial. Ultimately, this assignment is possible considering the correlations found in the ^15^N,^1^H-HMBC spectra. Thus, for instance, in compound **5b** the singlet signal due to H-5 (δ 8.32 ppm) exhibits a correlation to the ^15^N signal with δ -188.0 ppm, which consecutively must be that of N-7. The latter is also connected to the Ph H-2,6 resonance at δ 7.82 ppm, which accordingly has to be part of the N-7-phenyl system and thus can be unambiguously distinguished from Ph H-2,6 of the N1-phenyl moiety (δ 7.81 ppm). On basis of COSY (TOCSY), HSQC and HMBC experiments then the unequivocal assignment of all proton and carbon signals due to N1-Ph and N7-Ph is possible.

Compounds **4x** and **5x**, bearing no substituent at N-7, are interesting compounds capable of prototropic tautomerism with the 7*H*-, 6*H*- and XH-forms being possible (Scheme 3). The presence of XH-forms is improbable considering the chemical shifts for C-4 (**4x**: 170.5 ppm; **5x**: 193.5 ppm) which perfectly match those for δ (C-4) of all other N-7 substituted congeners of type **4** and **5**, with the latter having no possibility for the formation XH-isomers. As irradiation of the resonance due to the pyrazole NH proton gives the H-5 singlet a strong NOE (Scheme 3) a significant contribution of the 6*H*-form to the overall tautomeric composition is evident. This assumption is supported by ^13^C chemical shift data and by the size of certain ^13^C,^1^H coupling constants. Hence, in **5x**^2^*J*(C4a,H5) (7.8 Hz) is somewhat smaller than ^2^*J*(C3a,H3) (9.5 Hz) on the opposite site of the molecule what can be explained by lone-pair effects according to lit. [38]. Also ^3^*J*(C8a,H3) = 4.9 Hz markedly differs from the corresponding coupling constant ^3^*J*(C7a,H5) = 8.5 Hz. Moreover, δ C-7a (155.0 ppm) is larger than δ C-8a (147.6 ppm) and, conversely, δ C-5 (130.2 ppm) is significantly smaller than δ C-3 (137.7 ppm). Both phenomena can smoothly be explained by a strong contribution of the 6*H*-form in which C-5 is of ‘pyrazole C-5 type’ and C-7a of ‘pyrazole C-3 type’ – just opposite as for the 7*H*-form and for ‘fixed’ forms with a substituent attached to N-7. For comparison, δ (C-3) in 3-methoxy-1-phenylpyrazole (166.7 ppm) is markedly larger than δ (C-5) in 5-methoxy-1-phenylpyrazole (155.5 ppm), whereas – vice versa – δ (C-5) in 3-methoxy-1-phenylpyrazole (129.7 ppm) is significantly smaller than δ (C-3) in 5-methoxy-1-phenylypyrazole (139.6 ppm) [37,38,39].

Lastly, the spectra of esters **8a** and **8b** are characterized by the occurence of a pyrazole C−H moiety with typically small chemical shifts for pyrazole H-4 (**8a**: 6.04 ppm, **8b**: 6.30 ppm) and pyrazole C-4 (**8a**: 93.9 ppm, **8b**: 94.9 ppm). Compared to the corresponding signals in 4-aroylpyrazol-5-ols **3** (~160 ppm) the resonance of pyrazole C-5 in the O-substituted pyrazole units of compounds **8** are significantly shifted upfield (**8a**: 144.6 ppm, **8b**: 144.3 ppm). Furthermore, the C=O resonances of compounds **8** exhibit remarkably small chemical shifts typical for ester carbonyl C-atoms of aryl esters (**8a**: 157.3 ppm, **8b**: 157.2 ppm). 

## 3. Experimental

### 3.1. General

Melting points were determined on a Reichert–Kofler hot-stage microscope and are uncorrected. Mass spectra were obtained on a Shimadzu QP 1000 instrument (EI, 70 eV), a Finnigan MAT 8230 instrument (EI, 70 eV, HRMS), and a Finnigan MAT 900S instrument (ESI, 4 kV, MeOH-acetonitrile). IR spectra (KBr unless otherwise stated) were recorded on a Perkin-Elmer FT-IR 1605 spectrophotometer. Elemental analyses (C, H, N and S) were performed at the Microanalytical Laboratory, University of Vienna, and were in good agreement (±0.4%) with the calculated values. ^1^H- and ^13^C-NMR spectra were recorded on a Varian UnityPlus 300 spectrometer at 28 ºC (299.95 MHz for ^1^H, 75.43 MHz for ^13^C) or on a Bruker Avance 500 spectrometer at 293 K (500.13 MHz for ^1^H, 125.77 MHz for ^13^C). The center of the solvent signal was used as an internal standard, which was related to TMS with δ 7.26 ppm (^1^H, CDCl_3_), δ 2.49 ppm (^1^H, DMSO-*d*_6_), δ 77.0 ppm (^13^C, CDCl_3_) and δ 39.5 ppm (^13^C, DMSO-*d*_6_). ^15^N-NMR spectra (50.68 MHz) were obtained on a Bruker Avance 500 spectrometer with a ‘directly’ detecting broadband observe probe (BBFO) and were referenced against external nitromethane. The digital resolution was 0.25 Hz/data point in the ^1^H spectra and 0.4 Hz/data point in the ^13^C-NMR spectra. Systematic names were generated with ACD/Name [40] according to the IUPAC recommendations and were checked manually [41]. For chromatographic separations, Kieselgel 60 (70–230 mesh, Merck) was used. 

### 3.2. Synthetic procedures

#### 3.2.1. General procedure for the synthesis of the carbaldehydes **6a** and **6b**

Under anhydrous conditions, POCl_3_ (53.65 g, 32.5 mL, 350 mmol) was carefully added dropwise to dry DMF (11.70 g, 12.3 mL, 160 mmol) under cooling. Then pyrazolone **1** (50 mmol) was added and the mixture was heated to reflux for 2 hours. The reaction mixture was subsequently cooled to room temperature and the darkly coloured solution was poured onto ice water (approximately 300 mL) while stirring. After 30 minutes the precipitate formed was filtered off, washed with H_2_O and dried. 

*5-Chloro-1-phenyl-1H-pyrazole-4-carbaldehyde* (**6a**)*.* Starting from 1-phenyl-2-pyrazolin-5-one (**1a**, 8.01 g, 50 mmol) 8.69 g (84%) of compound **6a** were obtained as brownish crystals; m.p. 68 ºC (lit. [42] m.p. 70 ºC); ^1^H-NMR (500 MHz, CDCl_3_): δ (ppm) 9.92 (s, 1H, CHO), 8.14 (s, 1H, H-3), 7.46–7.57 (m, 5H, Ph-H); ^13^C-NMR (125 MHz, CDCl_3_): δ (ppm) 182.9 (CHO, ^1^*J* = 177.9 Hz, ^3^*J* (CHO,H-3) = 0.6 Hz), 140.9 (C-3, ^1^*J* (C-3,H-3) = 193.1 Hz, ^3^*J*(C-3,CHO) = 3.7 Hz), 136.8 (Ph C-1), 132.4 (C-5, ^3^*J*(C-5,H-3) = 5.9 Hz), 129.4 (Ph C-4), 129.3 (Ph C-3,5), 125.1 (Ph C-2,6), 120.1 (C-4, ^2^*J* (C-4,H-3) = 9.5 Hz, ^2^*J*(C-4,CHO) = 25.2 Hz); ^15^N-NMR (50 MHz, CDCl_3_): δ (ppm) −161.5 (N-1), −70.3 (N-2); MS *m/z* (%): 206/208 (M^+^, 89/30), 205/207 (M^+^-1, 93/38), 167 (33), 149 (100), 77 (75), 57 (46), 51 (58), 43 (36), 41 (42).

*5-Chloro-3-methyl-1-phenyl-1H-pyrazole-4-carbaldehyde* (**6b**). Starting from 3-methyl-1-phenyl-2-pyrazolin-5-one (**1b**, 8.71 g, 50 mmol) 9.05 g (82%) of compound **6b** were obtained as brownish crystals; m.p. 146 ºC (lit. [43] m.p. 146–147 ºC); ^1^H-NMR (500 MHz, CDCl_3_): δ (ppm) 9.96 (s, 1H, CHO), 7.53 (m, 2H, Ph H-2,6), 7.51 (m, 2H, Ph H-3,5), 7.46 (m, 1H, Ph H-4), 2.53 (s, 3H, Me); ^13^C-NMR (125 MHz, CDCl_3_): δ (ppm) 183.8 (CHO, ^1^*J* = 176.2 Hz), 151.7 (C-3, ^2^*J*(C-3,Me) = 7.0 Hz, ^3^*J*(C-3,CHO) = 4.6 Hz), 136.9 (Ph C-1), 133.4 (C-5), 129.2 (Ph C-3,5), 129.1 (Ph C-4), 125.1 (Ph C-2,6), 117.3 (C-4, ^2^*J*(C-4,CHO) = 24.3 Hz, ^3^*J*(C-4,Me) = 2.5 Hz), 13.8 (Me, ^1^*J* = 129.4 Hz); ^15^N-NMR (50 MHz, CDCl_3_): −168.1 (N-1), −76.1 (N-2).

#### 3.2.2. Preparation of *5-chloro-1-phenyl-1H-pyrazole-4-carboxylic acid* (**7a**)

To a solution of **6a** (1.03 g, 5 mmol) in a mixture of H_2_O/*t*-butanol 1:1 (15 mL) 1.11 g (7 mmol) KMnO_4_ in H_2_O (20 mL) was added dropwise over 3 h while stirring at 70–80 ºC. Then an aqueous solution of 10% KOH was added while stirring until the solution turned alkaline. The mixture was filtered, then the filtrate was acidified with concentrated hydrochloric acid to pH 2. The precipitated solid was filtered off, washed with water and dried. Yield: 970 mg (88%) of colorless crystals; m.p. 188 ºC (lit. [44] m.p. 187–188 ºC); ^1^H-NMR (300 MHz, DMSO-*d*_6_): δ (ppm) 12.97 (s, 1H, OH), 8.16 (s, 1H, H-3), 7.52–7.60 (m, 5H, Ph-H); ^13^C-NMR (75 MHz, DMSO-*d*_6_): δ (ppm) 162.3 (C=O), 142.4 (C-3, ^1^*J*(C-3,H-3) = 193.5 Hz), 137.2 (Ph-C-1), 130.2 (C-5, ^3^*J*(C-5,H-3) = 5.8 Hz), 129.3 (Ph C-3,4,5), 125.7 (Ph C-2,6), 112.3 (C-4, ^2^*J*(C-4,H-3) = 8.9 Hz); ^15^N-NMR (50 MHz, DMSO-*d*_6_): δ (ppm) −160.8 (N-1), −70.2 (N-2). MS *m/z* (%): 222/224 (M^+^, 83/27), 205 (32), 104 (19), 77 (100), 51 (92), 50 (28), 45 (26).

#### 3.2.3. Preparation of *5-chloro-3-methyl-1-phenyl-1H-pyrazole-4-carboxylic acid* (**7b**)

The title compound was prepared according to a related procedure given in [23].

#### 3.2.4. General procedure for the synthesis of the acid chlorides **2a** and **2b**

A suspension of the accordant carboxylic acid **7** (2 mmol) in toluene (10 mL), excess SOCl_2_ (10 mL) and 1 droplet of DMF was refluxed for 3 h. Then toluene and excess SOCl_2_ were distilled off. More toluene was added and the solvent was distilled off again. The remaining acid chloride was used immediately.

*5-Chloro-1-phenyl-1H-pyrazole-4-carbonyl chloride* (**2a**)*.* Starting from **7a** (445 mg, 2 mmol) 470 mg (98%) of compound **2a** were obtained as yellowish crystals; m.p. 134 ºC; ^1^H-NMR (500 MHz, CDCl_3_): δ (ppm) 8.25 (s, 1H, H-3), 7.54 (m, 5H, Ph-H); ^13^C-NMR (125 MHz, CDCl_3_): δ (ppm) 158.3 (C=O), 144.9 (C-3, ^1^*J*(C-3,H-3) = 196.2 Hz), 136.8 (Ph C-1), 132.2 (C-5, ^3^*J*(C-5,H-3) = 4.5 Hz), 129.8 (Ph C-4), 129.4 (Ph C-3,5), 125.3 (Ph C-2,6), 116.1 (C-4, ^2^*J*(C-4,H-3) = 8.8 Hz); ^15^N-NMR (50 MHz, CDCl_3_): δ (ppm) −157.7 (N-1), −71.0 (N-2); MS *m/z* (%): 240/242/244 (M^+^, 12/8/1), 205 (100), 77 (51), 51 (36). HRMS Calcd. for C_10_H_6_Cl_2_N_2_O: 239.9857. Found: 239.9851.

*5-Chloro-3-methyl-1-phenyl-1H-pyrazole-4-carbonyl chloride* (**2b**). Starting from **7b** (473 mg, 2 mmol) 481 mg (92%) of compound **2b** were obtained as a colorless powder; m.p. 97 ºC (lit. [45] m.p. 87 ºC); ^1^H-NMR (125 MHz, CDCl_3_): δ (ppm) 7.52 (m, 5H, Ph-H), 2.57 (s, 3H, 3-Me); ^13^C-NMR (125 MHz, CDCl_3_): δ (ppm) 158.4 (COCl), 151.9 (C-3, ^2^*J*(C-3,3-Me) = 7.1 Hz), 136.0 (Ph C-1), 132.2 (C-5), 128.6 (Ph C-4), 128.3 (Ph C-3,5), 124.6 (Ph C-2,6), 113.2 (C-4, ^3^*J*(C-4,3-Me) = 2.5 Hz), 14.5 (Me, ^1^*J* = 129.9 Hz); ^15^N-NMR (50 MHz, CDCl_3_): δ (ppm) −164.9 (N-1), −76.3 (N-2); IR: 1740 (C=O) cm^-1^; MS *m/z* (%): 254/256/258 (M^+^, 8/5/1), 219 (100), 155 (19), 91 (16), 77 (34), 51 (24).

#### 3.2.5. Acylation of Pyrazolones: General procedure for the synthesis of **3a-g** and **8a-b**

A solution of the appropriate acid chloride **2** (5 mmol) in dry 1,4-dioxane (5 mL) was added dropwise to a suspension of pyrazolone (**1a**-**e**, 5 mmol) and Ca(OH)_2_ (10 mmol) in dry 1,4-dioxane (5 mL). The reaction mixture was heated to reflux for 3 h under anhydrous conditions. After cooling to room temperature the mixture was treated with 2 N HCl (20 mL), stirred for 30 min and afterwards H_2_O (20 mL) was added. Then the products were filtered off, washed with H_2_O and recrystallized. Spectroscopic and analytical data of **3a**-**g** and **8a**-**b** are summarized in Table 1.

*(5-Chloro-1-phenyl-1H-pyrazol-4-yl)(5-hydroxy-1-phenyl-1*H*-pyrazol-4-yl)methanone* (**3a**). Starting from pyrazolone **1a** (801 mg, 5 mmol) and acid chloride **2a** (1.21 g, 5 mmol) 1.58 g (86%) of compound **3a** were obtained as yellowish crystals of m.p. 207 ºC (EtOH). 

*(5-Chloro-3-methyl-1-phenyl-1H-pyrazol-4-yl)(5-hydroxy-1-phenyl-1*H*-pyrazol-4-yl)methanone* (**3b**). Starting from pyrazolone **1a** (801 mg, 5 mmol) and acid chloride **2b** (1.28 g, 5 mmol) 1.04 g (55%) of compound **3b** were obtained as colorless crystals of m.p. 153 ºC (EtOH). 

*(5-Chloro-1-phenyl-1H-pyrazol-4-yl)(5-hydroxy-3-methyl-1-phenyl-1*H*-pyrazol-4-yl)methanone* (**3c**). Starting from pyrazolone **1b** (871 mg, 5 mmol) and acid chloride **2a** (1.21 g, 5 mmol) 1.37 g (72%) of compound **3c** were obtained as brownish crystals of m.p. 168 ºC (EtOH).

*(5-Chloro-3-methyl-1-phenyl-1H-pyrazol-4-yl)(5-hydroxy-3-methyl-1-phenyl-1*H*-pyrazol-4-yl)methan-one* (**3d**). Starting from pyrazolone **1b** (871 mg, 5 mmol) and acid chloride **2b** (1.28 g, 5 mmol) 943 mg (48%) of compound **3d** were obtained as colorless crystals of m.p. 217–219 ºC (EtOH) (lit. [29] m.p. 219–220 ºC).

*(5-Chloro-1-phenyl-1H-pyrazol-4-yl)(5-hydroxy-1,3-dimethyl-1*H*-pyrazol-4-yl)methanone* (**3e**). From pyrazolone **1c** (561 mg, 5 mmol) and acid chloride **2a** (1.21 g, 5 mmol) 1.03 g (65%) of compound **3e** were obtained as colorless crystals of m.p. 184 ºC (EtOH). 

*(5-Chloro-1-phenyl-1H-pyrazol-4-yl)[5-hydroxy-1-(4-methoxybenzyl)-1H-pyrazol-4-yl]methanone* (**3f**). Starting from pyrazolone **1d** (1.02 g, 5 mmol) and acid chloride **2a** (1.21 g, 5 mmol) 1.40 g (68%) of compound **3f** were obtained as colorless crystals of m.p. 166–167 ºC (EtOH). 

*(1-Benzyl-5-hydroxy-3-methyl-1H-pyrazol-4-yl)(5-chloro-1-phenyl-1*H*-pyrazol-4-yl)methanone* (**3g**). Starting from pyrazolone **1e** (941 mg, 5 mmol) and acid chloride **2a** (1.21 g, 5 mmol) 1.92 g (98%) of compound **3g** were obtained as orange crystals of m.p. 119 ºC (EtOH). 

*1,3-Dimethyl-1H-pyrazol-5-yl 5-chloro-3-methyl-1-phenyl-1*H*-pyrazole-4-carboxylate* (**8a**). Starting from pyrazolone **1c** (561 mg, 5 mmol) and acid chloride **2b** (1.28 g, 5 mmol) 810 mg (49%) of compound **8a** were obtained as colorless crystals of m.p. 146 ºC (EtOH).

*1-(4-Methoxybenzyl)-1H-pyrazol-5-yl 5-chloro-3-methyl-1-phenyl-1*H*-pyrazole-4-carboxylate* (**8b**). Starting from pyrazolone **1d** (1.021 g, 5 mmol) and acid chloride **2b** (1.28 g, 5 mmol) 1.08 g (51%) of compound **8b** were obtained as colorless crystals of m.p. 116 ºC (EtOH). 

**Table 1:** Spectroscopic and analytical data of compounds **3a**-**g** and **8a**-**b**.

Entry	Structure	Spectroscopic and analytical data
**3a**	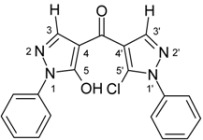	^1^H-NMR (300 MHz, CDCl_3_): δ (ppm) 8.26 (s, 1H, H-3’), 8.02 (s, 1H, H-3), 7.89 (m, 2H, N1-Ph H-2,6), 7.54–7.58 (3H, N1’-Ph H-3,4,5), 7.57 (m, 2H, N1’-Ph H-2,6), 7.50 (m, 2H, N1-Ph H-3,5), 7.35 (m, 1H, N1-Ph H-4), 6.0–8.5 (very broad s, 1H, OH); ^13^C-NMR (75 MHz, CDCl_3_): δ (ppm) 181.5 (C=O), 159.9 (C-5, ^3^*J*(C-5,H-3) = 4.9 Hz), 141.1 (C-3’, ^1^*J*(C-3’,H-3’) = 190.7 Hz), 138.5 (C-3, ^1^*J*(C-3,H-3) = 189.3 Hz), 137.2 (N1-Ph C-1 and N-1’-Ph C-1), 130.5 (C-5’, ^3^*J*(C-5’,H-3’) = 5.9 Hz), 129.5 (N1’-Ph C-4), 129.3 (N1’-Ph C-3,5), 129.2 (N1-Ph C-3,5), 127.2 (N1-Ph C-4), 125.5 (N1’-Ph C-2,6), 121.1 (N1-Ph C-2,6), 117.9 (C-4’, ^2^*J*(C-4’,H-3’) = 10.3 Hz), 103.7 (C-4, ^2^*J*(C-4,H-3) = 11.1 Hz); ^15^N-NMR (50 MHz, CDCl_3_): δ (ppm) −185.2 (N-1), −159.9 (N-1’), −97.9 (N-2), −72.4 (N-2’); IR: 1656 (C=O) cm^-1^; MS *m/z* (%): 364/266 (M^+^, 23/8), 329 (38), 186 (100), 91 (18), 77 (52), 51 (22). Calcd. for C_19_H_13_ClN_4_O_2_ (364.79): C, 62.56; H, 3.59; N, 15.36. Found: C, 62.39; H, 3.50; N, 15.16.
**3b**	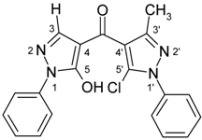	^1^H-NMR (500 MHz, CDCl_3_): δ (ppm) 10.18 (broad s, 1H, OH), 7.92 (s, 1H, H-3), 7.89 (m, 2H, N1-Ph H-2,6), 7.58 (m, 2H, N1’-Ph H-2,6), 7.53 (m, 2H, N1’-Ph H-3,5), 7.49 (m, 2H, N1-Ph H-3,5), 7.48 (1H, N1’-Ph H-4), 7.34 (m, 1H, N1-Ph H-4), 2.50 (s, 3H, 3’-Me); ^13^C-NMR (125 MHz, CDCl_3_): δ (ppm) 183.0 (C=O), 160.0 (C-5, ^3^*J*(C-5,H-3) = 4.7 Hz), 150.5 (C-3’, ^2^*J*(C-3’,3’-Me) = 6.9 Hz), 140.1 (C-3, ^1^*J*(C-3,H-3) = 191.1 Hz), 137.3 (N1’-Ph C-1), 137.2 (N1-Ph C-1), 129.2 (N1’-Ph C-3,5), 129.15 (N1-Ph C-3,5), 129.05 (N1’-Ph C-4), 127.6 (C-5’), 127.0 (N1-Ph C-4), 125.4 (N1’-Ph C-2,6), 120.9 (N1-Ph C-2,6), 117.4 (C-4’, ^3^*J*(C-4’,3’-Me) = 2.8 Hz), 104.2 (C-4, ^2^*J*(C-4,H-3) = 10.6 Hz), 13.8 (3’-Me, ^1^*J* = 129.1 Hz); ^15^N-NMR (50 MHz, CDCl_3_): δ (ppm) −186.0 (N-1), −167.8 (N-1’), −97.3 (N-2), −75.8 (N-2’); IR: 1559 (C=O) cm^-1^; MS *m/z* (%): 378/380 (M^+^, 12/4), 343 (45), 219 (11), 186 (100), 118 (12), 91 (15), 77 (43), 51 (16). Calcd. for C_20_H_15_ClN_4_O_2_ (378.81): C, 63.41; H, 3.99; N, 14.79. Found: C, 63.24; H, 3.81; N, 14.74.
**3c**	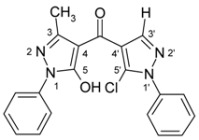	^1^H-NMR (500 MHz, CDCl_3_): δ (ppm) 8.01 (s, 1H, H-3’), 7.87 (m, 2H, N1-Ph H-2,6), 7.60 (m, 2H, N1’-Ph H-2,6), 7.55 (m, 2H, N1’-Ph H-3,5), 7.52 (m, 1H, N1’-Ph H-4), 7.48 (m, 2H, N1-Ph H-3,5), 7.32 (m, N1-Ph H-4), 2.39 (s, 3H, 3-Me), OH not found; ^13^C-NMR (125 MHz, CDCl_3_): δ (ppm) 182.9 (C=O), 161.0 (C-5), 147.3 (C-3, ^2^*J*(C-3,3-Me) = 6.8 Hz), 140.8 (C-3’, ^1^*J*(C-3’,H-3’) = 192.0 Hz), 137.3 (N1’-Ph C-1), 137.1 (N1-Ph C-1), 129.3 (N1’-Ph C-3,4,5), 129.1 (N1-Ph C-3,5), 129.0 (C-5’, ^3^*J*(C-5’,H-3’) = 5.6 Hz), 126.9 (N1-Ph C-4), 125.4 (N1’-Ph C-2,6), 120.9 (N1-Ph C-2,6), 118.4 (C-4’, ^2^*J*(C-4’,H-3’) = 10.1 Hz), 104.4 (C-4, ^3^*J*(C-4,3-Me) = 2.7 Hz), 15.6 (3-Me, ^1^*J* = 128.8 Hz); ^15^N-NMR (50 MHz, CDCl_3_): δ (ppm) −190.3 (N-1), −161.7 (N-1’), −100.8 (N-2), −74.2 (N-2’); IR: 1619 (C=O) cm^-1^; MS *m/z* (%): 378/380 (M^+^, 8/3), 342 (48), 200 (100), 91 (37), 77 (58), 51 (25). Calcd. for C_20_H_15_ClN_4_O_2_ (378.81): C, 63.41; H, 3.99; N, 14.79. Found: C, 63.71; H, 3.91; N, 14.69.
**3d**	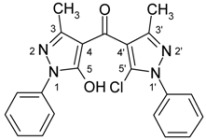	^1^H-NMR (500 MHz, CDCl_3_): δ (ppm) 9.20 (broad s, 1H, OH), 7.87 (m, 2H, N1-Ph H-2,6), 7.56 (m, 2H, N1’-Ph H-2,6), 7.52 (m, 2H, N1’-Ph H-3,5), 7.47 (m, 2H, N1-Ph H-3,5), 7.47 (m, 1H, N1’-Ph H-4), 7.31 (m, 1H, N1-Ph H-4), 2.41 (s, 3H, 3’-Me), 2.23 (s, 3H, 3-Me); ^13^C-NMR (125 MHz, CDCl_3_): δ (ppm) 183.8 (C=O), 160.8 (C-5), 148.8 (C-3’, ^2^*J*(C-3’,3’-Me) = 6.8 Hz), 148.1 (C-3, ^2^*J*(C-3,3-Me) = 6.7 Hz), 137.4 (N1’-Ph C-1), 137.1 (N1-Ph C-1), 129.2 (N1’-Ph C-3,5), 129.1 (N1-Ph C-3,5), 128.9 (N1’-Ph C-4), 126.8 (N1-Ph C-4), 126.7 (C-5’), 125.2 (N1’-Ph C-2,6), 120.7 (N1-Ph C-2,6), 117.8 (C-4’, ^3^*J*(C-4’,3’-Me) = 3.0 Hz), 105.5 (C-4, ^3^*J*(C-4,3-Me) = 2.8 Hz), 13.9 (3-Me, ^1^*J* = 129.0 Hz), 13.0 (3’-Me, ^1^*J* = 128.9 Hz); ^15^N-NMR (50 MHz, CDCl_3_): δ (ppm) −190.3 (N-1), −168.8 (N-1’), −100.0 (N-2), −76.6 (N-2’); MS *m/z* (%): 392/394 (M^+^, 5/2), 356 (39), 219 (10), 200 (100), 132 (13), 91 (31), 77 (40), 67 (12), 51 (15). Calcd. for C_21_H_17_ClN_4_O_2_ (392.84): C, 64.21; H, 4.36; N, 14.26. Found: C, 64.04; H, 4.18; N, 14.21.
**3e**	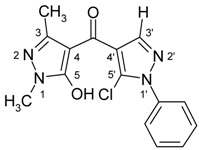	^1^H-NMR (300 MHz, CDCl_3_): δ (ppm) 10.35 (broad s, 1H, OH), 7.94 (s, 1H, H-3’), 7.44–7.59 (m, 5H, N1’-Ph), 3.62 (s, 3H, N1-Me), 2.27 (s, 3H, 3-Me); ^13^C-NMR (75 MHz, CDCl_3_): δ (ppm) 183.4 (C=O), 160.2 (C-5, ^3^*J*(C-5,N1-Me) = 2.3 Hz), 146.2 (C-3, ^2^*J*(C-3,3-Me) = 6.9 Hz), 140.6 (C-3’, ^1^*J(*C-3’,H-3’) = 191.7 Hz), 137.4 (N1’-Ph C-1), 129.2 (N1’-Ph C-3,4,5), 128.6 (C-5’, ^3^*J*(C-5’,H-3’) = 5.7 Hz), 125.3 (N1’-Ph C-2,6), 119.0 (C-4’, ^2^*J*(C-4’,H-3’) = 10.1 Hz), 103.2 (C-4, ^3^*J*(C-4,3-Me) = 2.7 Hz), 32.5 (N1-Me, ^1^*J* = 140.9 Hz), 15.2 (3-Me, ^1^*J* = 128.6 Hz); ^15^N-NMR (50 MHz, CDCl_3_): δ (ppm) −207.9 (N-1), −162.2 (N-1’), −100.5 (N-2), −74.7 (N-2’); IR: 1636 (C=O) cm^-1^;MS *m/z* (%): 316/318 (M^+^, 11/4), 281 (26), 138 (100), 77 (21), 51 (17). Calcd. for C_15_H_13_ClN_4_O_2_ (316.74): C, 56.88; H, 4.14; N, 17.69. Found: C, 57.12; H, 3.97; N, 17.61.
**3f**	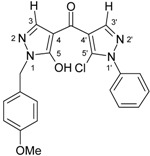	^1^H-NMR (300 MHz, CDCl_3_): δ (ppm) 8.18 (s, 1H, H-3’), 7.30–8.00 (very broad s, 1H, OH), 7.82 (broad, s, 1H, H-3), 7.55 (m, 2H, N1’-Ph H-2,6), 7.52 (m, 3H, N1’-Ph H-3,4,5), 7.31 (m, 2H, CH_2_-Ph H-2,6), 6.88 (CH_2_-Ph H-3,-5), 5.13 (broad s, 2H, CH_2_), 3.79 (s, 3H, OMe); ^13^C-NMR (75 MHz, CDCl_3_): δ (ppm) 181.7 (C=O), 159.5 (CH_2_-Ph C-4), 159.0 (C-5), 141.1 (C-3’, ^1^*J*(C-3’,H-3’) = 190.7 Hz), 137.9 (C-3, ^1^*J*(C-3,H-3) = 188.8 Hz), 137.3 (N1’-Ph C-1), 130.3 (C-5’), 129.6 (CH_2_-Ph C-2,6), 129.4 (N1’-Ph C-4), 129.2 (N1’-Ph C-3,5), 127.5 (CH_2_-Ph C-1), 125.5 (N1’-Ph C-2,-6), 118.2 (C-4’, ^2^*J*(C-4’,H-3’) = 10.0 Hz), 114.2 (CH_2_-Ph C3,5), 103.1 (C-4), 55.3 (OMe, ^1^*J* = 143.9 Hz), 49.8 (CH_2_, ^1^*J* = 140.6 Hz); ^15^N-NMR (50 MHz, CDCl_3_): δ (ppm) −189.2 (N-1), −160.3 (N-1’), −98.3 (N-2), −73.1 (N-2’); IR: 1621 (C=O) cm^-1^;MS *m/z* (%): 408/410 (M^+^, 7/2), 373 (25), 121 (100), 77 (23). Calcd. for C_21_H_17_ClN_4_O_3_ (408.84): C, 61.69; H, 4.19; N, 13.70. Found: C, 61.47; H, 4.13, N, 13.55.
**3g**	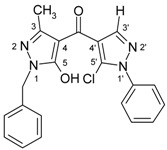	^1^H-NMR (300 MHz, CDCl_3_): δ (ppm) 7.70–8.20 (broad s, 1H, OH), 7.96 (s, 1H, H-3’), 7.58 (m, 2H, N1’-Ph H-2,6), 7.53 (m, 2H, N1’-Ph H-3,5), 7.48 (m, 1H, N1’-Ph H-4), 7.28–7.38 (m, 5H, CH_2_-Ph), 5.12 (s, 2H, CH_2_), 2.28 (s, 3H, 3-Me); ^13^C-NMR (75 MHz, CDCl_3_): δ (ppm) 183.3 (C=O), 160.3 (C-5, ^3^*J*(C-5,CH_2_) = 2.4 Hz), 146.6 (C-3, ^2^*J*(C-3,3-Me) = 7.0 Hz), 140.7 (C-3’, ^1^*J*(C-3’,H-3’) = 192.0 Hz), 137.4 (N1’-Ph C-1), 135.5 (CH_2_-Ph C-1), 129.2 (N1’-Ph C-3,4,5), 128.7 (CH_2_-Ph C-3,5 and C-5’, ^3^*J*(C-5’,H-3’) = 5.7 Hz), 128.0 (CH_2_-Ph C-2,4,6), 125.3 (N1’-Ph C-2,6), 118.9 (C-4’, ^2^*J*(C-4’,H-3’) = 10.1 Hz), 103.4 (C-4, ^3^*J*(C-4,3-Me) = 2.6 Hz), 49.8 (CH_2_, ^1^*J* = 140.2 Hz), 15.4 (3-Me, ^1^*J* = 128.6 Hz); ^15^N-NMR (50 MHz, CDCl_3_): δ (ppm) −196.4 (N-1), −162.1 (N-1’), −101.1 (N-2), −74.7 (N-2’); IR: 1633 (C=O) cm^-1^; MS *m/z* (%): 392/394 (M^+^, 8/3), 356 (91), 91 (100), 77 (25), 51 (18). Calcd. for C_21_H_17_ClN_4_O_2_ (392.84): C, 64.21; H, 4.36; N, 14.26. Found: C, 64.13; H, 4.27; N, 14.11.
**8a**	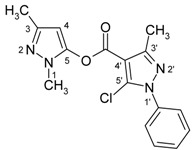	^1^H-NMR (300 MHz, CDCl_3_): δ (ppm) 7.52 (m, 5H, Ph-H), 6.04 (s, 1H, H-4), 3.74 (s, 3H, N-Me), 2.59 (s, 3H, 3’-Me), 2.25 (s, 3H, 3-Me); ^13^C-NMR (75Hz, CDCl_3_): δ (ppm) 157.3 (C=O), 153.1 (C-3’, ^2^*J*(C-3’,3’-Me) = 6.9 Hz), 147.2 (C-3, ^2^*J*(C-3,3-Me) = 6.7 Hz, ^2^*J*(C-3,H-4) = 4.3 Hz), 144.6 (C-5, ^3^*J*(C-5,N-CH_3_) = 2.2 Hz, ^2^*J*(C 5,H-4) = 4.4 Hz), 137.2 (Ph-C-1), 132.2 (C-5’), 129.3 (Ph-C-4), 129.2 (Ph-C-3,5), 125.5 (Ph-C-2,-6), 108.3 (C-4’, ^3^*J*(C-4’,3’-Me) = 2.7 Hz), 93.9 (C-4, ^1^*J*(C-4,H-4) = 181.2 Hz, ^3^*J*(C-4,3-Me) = 3.5 Hz), 14.8 (3’-Me, ^1^*J* = 129.5Hz), 14.2 (3-Me, ^1^*J* = 127.5 Hz); ^15^N-NMR (50 MHz, CDCl_3_): δ (ppm) −202.2 (N-1), −165.8 (N-1’), −98.9 (N-2), −75.7 (N-2’); IR: 1745 (C=O) cm^-1^; MS *m/z* (%): 330 (M^+^, 0.1), 219 (100), 77 (42), 51 (19). Calcd. for C_16_H_15_ClN_4_O_2_ (330.77): C, 58.10; H, 4.57; N, 16.94. Found: C, 58.14; H, 4.37; N, 16.90.
**8b**	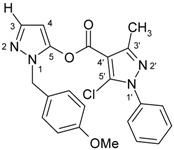	^1^H-NMR (300 MHz, CDCl_3_): δ (ppm) 7.53 (d, 1H, H-3, ^3^*J*(H3,H4) = 2.1 Hz), 7.52 (m, 5H, N1’-Ph), 7.12 (m, 2H, CH_2_-Ph H-2,6), 6.82 (m, 2H, CH_2_-Ph H-3,5), 6.30 (d, 1H, H-4, ^3^*J*(H4,H3) = 2.1 Hz), 5.28 (s, 2H, CH_2_), 3.75 (s, 3H, O-Me), 2.52 (s, 3H, 3’-Me); ^13^C-NMR (75 MHz, CDCl_3_): δ (ppm) 159.2 (CH_2_-Ph C4), 157.2 (C=O), 153.2 (C-3’, ^2^*J*(C-3’,3’-Me) = 7.0 Hz), 144.3 (C-5), 138.7 (C-3, ^1^*J*(C-3,H-3) = 187.6 Hz, ^2^*J*(C-3,H-4) = 4.8 Hz), 137.2 (N1’-Ph C-1), 132.2 (C-5’), 129.4 (N1’-Ph C-4), 129.2 (N1’-Ph C-3,5), 128.4 (CH_2_-Ph C-2,6), 128.3 (CH_2_-Ph C-1), 125.5 (N1’-Ph C-2,6), 114.1 (CH_2_-Ph C-3,5), 108.2 (C-4’, ^3^*J*(C-4’,3’-Me) = 2.7 Hz), 94.9 (C-4, ^1^*J*(C-4,H-4) = 183.4 Hz, ^2^*J*(C-4,H-5) = 10.5 Hz), 55.2 (O-Me, ^1^*J* = 143.8 Hz), 51.3 (CH_2_, ^1^*J* = 140.0 Hz, ^3^*J*(CH_2_,Ph-H-2,6) = 4.3 Hz), 14.8 (3’-Me, ^1^*J* = 129.6 Hz); ^15^N-NMR (50 MHz, CDCl_3_): δ (ppm) −185.1 (N-1), −165.7 (N-1’), −95.3 (N-2), −75.7 (N-2’); IR: 1745 (C=O) cm^-1^; MS *m/z* (%): 422 (M^+^, 0.1), 219 (100), 121 (25), 77 (20). Calcd. for C_22_H_19_ClN_4_O_3_ (422.86): C, 62.49; H, 4.53; N, 13.25. Found: C, 62.45; H, 4.40; N, 13.15.

#### 3.2.6. Cyclization of 4-Aroylpyrazolones **3a-g**: General procedure for the synthesis of **4a-b, 4d-g**

Under anhydrous conditions, K_2_CO_3_ (138 mg, 1 mmol) was added to a solution of the appropriate type **3**compound (1 mmol) in dry DMF (10 mL), then the mixture was heated to reflux for 2 h. After evaporation of the solvent, 20 mL of H_2_O were added to the residue. The precipitate was filtered off, washed with water and recrystallized from EtOH. NMR data of the products are given in Table 2, Table 3, Table 4 and Table 5.

*1,7-Diphenyl-1*H*-pyrano[2,3-*c*:6,5-*c*]dipyrazol-4(7H)-one* (**4a**). Starting from **3a** (365 mg, 1 mmol) 304 mg (93%) of compound **4a** were obtained as colorless crystals; m.p. 256 ºC (EtOH); IR: 1673 (C=O) cm^-1^; MS *m/z* (%): 328 (M^+^, 77), 187 (30), 77 (100), 51 (40). Calcd. for C_19_H_12_N_4_O_2_ (328.32): C, 69.51; H, 3.68; N, 17.06. Found: C, 69.36; H, 3.53; N, 16.84.

*3-Methyl-1,7-diphenyl-1H-pyrano[2,3-*c*:6,5-*c*]dipyrazol-4(7H)-one* (**4b**). Starting from **3b** (379 mg, 1 mmol) 288 mg (84%) of compound **4b** were obtained as colorless crystals. Alternatively, starting from **3c** (379 mg, 1 mmol) 219 mg (64%) of compound **4b** were obtained as colorless crystals; m.p. 220 ºC (EtOH); IR: 1664 (C=O) cm^-1^; MS *m/z* (%): 342 (M^+^, 66), 156 (14), 91 (24), 77 (100), 67 (20), 51 (45). Calcd. for C_20_H_14_N_4_O_2_ (342.35): C, 70.14; H, 4.12; N, 16.37. Found: C, 69.93; H, 3.96; N, 16.34.

*3,5-Dimethyl-1,7-diphenyl-1H-pyrano[2,3-*c*:6,5-*c*]dipyrazol-4(7H)-one* (**4d**). Starting from **3d** (393 mg, 1 mmol) 264 mg (74%) of compound **4d** were obtained as colorless crystals; m.p. 243 ºC (EtOH) (lit. [29] m.p. 240–241 ºC). IR: 1521 (C=O) cm^-1^; MS *m/z* (%): 356 (M^+^, 100), 178 (10), 156 (18), 91 (31), 77 (99), 67 (30), 51 (41). Calcd. for C_21_H_16_N_4_O_2_ (356.37)•0.2 H_2_O: C, 70.07; H, 4.59; N, 15.72. Found: C, 69.96; H, 4.38; N, 15.56.

*1,3-Dimethyl-7-phenyl-1H-pyrano[2,3-*c*:6,5-*c*]dipyrazol-4(7H)-one* (**4e**). Starting from **3e** (317 mg, 1 mmol) 185 mg (66%) of compound **4e** were obtained as colorless crystals; m.p. 204 ºC (EtOH); IR: 1654 (C=O) cm^-1^; MS *m/z* (%): 280 (M^+^, 100), 139 (25), 77 (74), 51 (43). Calcd. for C_15_H_12_N_4_O_2_ (280.28): C, 64.28; H, 4.32; N, 19.99. Found: C, 64.17; H, 4.23; N, 19.82.

*1-(4-Methoxybenzyl)-7-phenyl-1H-pyrano[2,3-*c*:6,5-*c*]dipyrazol-4(7H)-one* (**4f**). Starting from **3f** (409 mg, 1 mmol) 276 mg (74%) of compound **4f** were obtained as colorless crystals; m.p. 245 ºC (EtOH); IR: 1667 (C=O) cm^-1^; MS *m/z* (%): 372 (M^+^, 20), 121 (100), 77 (15). Calcd. for C_21_H_16_N_4_O_3_ (372.38): C, 67.73; H, 4.33; N, 15.05. Found: C, 67.78; H, 4.18; N, 14.97.

*1-Benzyl-3-methyl-7-phenyl-1*H*-pyrano[2,3-*c*:6,5-*c*]dipyrazol-4-(7H)-one* (**4g**). Starting from **3g** (393 mg, 1 mmol) 258 mg (73%) of compound **4g** were obtained as colorless crystals; m.p. 210 ºC (EtOH); IR: 1659 (C=O) cm^-1^; MS *m/z* (%): 356 (M^+^, 39), 265 (33), 91 (100), 77 (28), 51 (14). Calcd. for C_21_H_16_N_4_O_2_ (356.38): C, 70.77; H, 4.53; N, 15.72. Found: C, 70.89; H, 4.34; N, 15.64.

#### 3.2.7. General procedure for the synthesis of **5a-b** and **5d-g**

Lawesson’s Reagent (202 mg, 0.5 mmol) was added to a solution of the appropriate oxo compound **4** in 15 mL of toluene and the mixture was heated to reflux for approx. 14 h. After cooling, the precipitate was filtered off and recrystallized from EtOH. In case of **5b** no precipitate was formed, here the solvent was evaporated and the residue was subjected to column chromatography (silica gel, mobile phase CH_2_Cl_2_:MeOH/9:1) in order to obtain the colored thione which was crystallized from EtOH for analytical purposes. NMR data of the products are given in Table 2, Table 3, Table 4 and Table 5.

*1,7-Diphenyl-1H-pyrano[2,3-*c*:6,5-*c*]dipyrazol-4(7H)-thione* (**5a**). Starting from **4a** (328 mg, 1 mmol) 204 mg (60%) of compound **5a** were obtained as reddish crystals; m.p. 254–256 ºC (EtOH); MS *m/z* (%): 344 (M^+^, 100), 201 (30), 77 (84), 51 (57). Calcd. for C_19_H_12_N_4_OS (344.39)•0.2 H_2_O: C, 65.58; H, 3.59; N, 16.10. Found: C, 65.57; H, 3.37; N, 15.73.

*3-Methyl-1,7-diphenyl-1H-pyrano[2,3-*c*:6,5-*c*]dipyrazol-4(7H)-thione* (**5b**). Starting from **4b** resp. **4c** (342 mg, 1 mmol) 356 mg (99%) of compound **5b** were obtained as orange crystals; m.p. 195–197 ºC (EtOH); MS *m/z* (%): 358 (M^+^, 22), 356 (100), 77 (50), 51 (25). Calcd. for C_20_H_14_N_4_OS (358.42): C, 67.02; H, 3.94; N, 15.63. Found: C, 67.03; H, 3.82; N, 15.57.

*3,5-Dimethyl-1,7-diphenyl-1H-pyrano[2,3-*c*:6,5-*c*]dipyrazol-4(7H)-thione* (**5d**). Starting from **4d** 356 mg, 1 mmol) 268 mg (72%) of compound **5d** were obtained as deep yellow crystals; m.p. 286 ºC (EtOH) (lit. [29] m.p. 285–286 ºC); MS *m/z* (%): 373 (M^+^+1, 23), 372 (M^+^, 100), 186 (11), 77 (46), 51 (27).

*1,3-Dimethyl-7-phenyl-1H-pyrano[2,3-*c*:6,5-*c*]dipyrazol-4(7H)-thione* (**5e**). Starting from **4e** (280 mg, 1 mmol) 138 mg (47%) of compound **5e** were obtained as yellowish crystals; m.p. 212 ºC (EtOH); MS *m/z* (%): 296 (M^+^, 18), 275 (42), 73 (100). Calcd. for C_15_H_12_N_4_OS (296.35): C, 60.79; H, 4.08; N, 18.91. Found: C, 60.77; H, 3.94; N, 18.58.

*1-(4-Methoxybenzyl)-7-phenyl-1H-pyrano[2,3-*c*:6,5-*c*]dipyrazol-4(7H)-thione* (**5f**). Starting from **4f**(372 mg, 1 mmol) 314 mg (81%) of compound **5f** were obtained as yellowish crystals; m.p. 222 ºC (EtOH); MS *m/z* (%): 388 (M^+^, 13), 121 (100), 91 (10), 77 (31), 51 (14). Calcd. for C_21_H_16_N_4_O_2_S (388.44)•0.2 H_2_O: C, 64.34; H, 4.22; N, 14.29. Found: C, 64.40; H, 3.96; N, 14.12.

*1-Benzyl-3-methyl-7-phenyl-1H-pyrano[2,3-*c*:6,5-*c*]dipyrazol-4-(7H)-thione* (**5g**). Starting from **4g** (356 mg, 1 mmol) 268 mg (72%) of compound **5g** were obtained as yellowish crystals; m.p. 224 ºC (EtOH); MS *m/z* (%): 373 (M^+^+1, 21), 372 (M^+^, 100), 281 (75), 91 (97), 77 (37), 51 (33). Calcd. for C_21_H_16_N_4_OS (372.44): C, 67.72; H, 4.33; N, 15.04. Found: C, 67.37; H, 4.15; N, 14.85.

#### 3.2.8. General procedure for the synthesis of **4x** and **5x**

Under anhydrous conditions, a solution of the PMB-substituted congener **4f** or **5f** (0.5 mmol) and trifluoroacetic acid (TFA, 1.43 g, 12.5 mmol) was heated to reflux overnight. After removal of excess TFA under reduced pressure the residue was stored over solid KOH. Then ice-cold diethyl ether-acetone (3:1) was added. The precipitate was filtered off and washed with cold diethyl ether-acetone. NMR data of the products are given in Table 2, Table 3, Table 4 and Table 5.

*1-Phenyl-1*H*-pyrano[2,3-*c*:6,5-*c*]dipyrazol-4(7*H*)-one* (**4x**). Starting from **4f** (186 mg, 0.5 mmol) 61 mg (48%) of compound **4x** were obtained as brown powder; m.p. 327–329 ºC; IR: 1681 (C=O) cm^-1^; MS *m/z* (%): 253 (M^+^+1, 16), 252 (M+, 100), 111 (87), 77 (46), 51 (38). HRMS Calcd. for C_13_H_8_N_4_O_2_: 252.0647. Found: 252.0644.

*1-Phenyl-1*H*-pyrano[2,3-*c*:6,5-*c*]dipyrazol-4(7*H*)-thione* (**5x**). Starting from **5f** (194 mg, 0.5 mmol) 69 mg (51%) of compound **5x** were obtained as a greenish powder; m.p. 271–273 ºC; MS *m/z* (%): 269 (M^+^+1, 18), 268 (M^+^, 100), 267 (M^+^-1, 45)127 (43), 77 (54), 51 (45). HRMS Calcd. for C_13_H_7_N_4_OS (M^+^-1): 267.0341. Found: 267.0335.

**Table 2:** ^1^H-NMR chemical shifts of **4a**-**b**, **4d**-**g**, **5a**-**b**, **5d-g**, **4x** and **5x** (δ in ppm).

Comp	Solvent	H of R^1^	H of R^3^	H of R^5^	H of R^7^
**4a**	CDCl_3_	Ph: 7.82 (2,6), 7.55 (3,5), 7.44 (4)	8.22 (H-3)	8.22 (H-5)	Ph: 7.82 (2,6), 7.55 (3,5), 7.44 (4)
**4b**	CDCl_3_	Ph: 7.78 (2,6), 7.52 (3,5), 7.40 (4)	2.66 (Me)	8.17 (H-5)	Ph: 7.80 (2,6), 7.53 (3,5), 7.43 (4)
**4d**	CDCl_3_	Ph: 7.78 (2,6), 7.51 (3,5), 7.39 (4)	2.65 (Me)	2.65 (Me)	Ph: 7.78 (2,6), 7.51 (3,5), 7.39 (4)
**4e**	CDCl_3_	3.87 (Me)	2.55 (Me)	8.13 (H-5)	Ph: 7.79 (2,6), 7.56 (3,5), 7.43 (4)
**4f**	CDCl_3_	Ph: 7.28 (2,6), 6.89 (3,5); 5.37 (CH_2_), 3.79 (OMe)	8.05 (H-3)	8.17 (H-5)	Ph: 7.66 (2,6), 7.56 (3,5), 7.46 (4)
**4g**	CDCl_3_	Ph: 7.37 (4), 7.36 (3,5), 7.31 (2,6); 5.35 (CH_2_)	2.60 (Me)	8.12 (H-5)	Ph: 7.60 (2,6), 7.52 (3,5), 7.42 (4)
**5a**	CDCl_3_	Ph: 7.84 (2,6), 7.57 (3,5), 7.46 (4)	8.39 (H-3)	8.39 (H-5)	Ph: 7.84 (2,6), 7.57 (3,5), 7.46 (4)
**5b**	CDCl_3_	Ph: 7.81 (2,6), 7.54 (3,5), 7.42 (4)	2.78 (Me)	8.32 (H-5)	Ph: 7.82 (2,6), 7.55 (3,5), 7.44 (4)
**5d**	CDCl_3_	Ph: 7.80 (2,6), 7.53 (3,5), 7.41 (4)	2.80 (Me)	2.80 (Me)	Ph: 7.80 (2,6), 7.53 (3,5), 7.41 (4)
**5e**	CDCl_3_	3.89 (Me)	2.65 (Me)	8.27 (H-5)	Ph: 7.81 (2,6), 7.57 (3,5), 7.45 (4)
**5f**	CDCl_3_	Ph: 7.28 (2,6), 6.90 (3,5); 5.38 (CH_2_), 3.79 (OMe)	8.20 (H-3)	8.32 (H-5)	Ph: 7.66 (2,6), 7.56 (3,5), 7.47 (4)
**5g**	CDCl_3_	Ph: 7.38 (3,4,5), 7.33 (2,6); 5.37 (CH_2_)	2.73 (Me)	8.29 (H-5)	Ph: 7.61 (2,6), 7.53 (3,5), 7.43 (4)
**4x**	DMSO- *d*_6_	Ph: 7.85 (2,6), 7.62 (3,5), 7.48 (4)	8.24 (H-3)	8.56 (H-5)	13.78 (NH)
**5x**	DMSO- *d*_6_	Ph: 7.87 (2,6), 7.64 (3,5), 7.50 (4)	8.34 (H-3)	8.66 (H-5)	14.00 (NH)

**Table 3:** ^13^C-NMR chemical shifts of **4a**-**b**, **4d**-**g**, **5a**-**b**, **5d**-**g**, **4x** and **5x** (δ in ppm, solvents as listed in Table 2).

Comp	C-3	C-3a	C-4	C-4a	C-5	C-7a	C-8a	C of R^1^	C of R^3^	C of R^5^	C of R^7^
**4a**	136.7	108.8	169.7	108.8	136.7	151.3	151.3	Ph: 136.6 (1), 129.6 (3,5), 128.3 (4), 121.3 (2,6)	-	-	Ph: 136.6 (1), 129.6 (3,5), 128.3 (4), 121.3 (2,6)
**4b**	148.1	106.5	170.6	108.9	136.6	151.3	151.4	Ph: 136.6 (1), 129.48 (3,5), 127.8 (4), 121.1 (2,6)	14.0 (Me)	-	Ph: 136.7 (1), 129.53 (3,5), 128.1 (4), 121.2 (2,6)
**4d**	148.0	106.6	171.8	106.6	148.0	151.5	151.5	Ph: 136.7 (1), 129.5 (3,5), 127.7 (4), 121.0 (2,6)	14.0 (Me)	14.0 (Me)	Ph: 136.7 (1), 129.5 (3,5), 127.7 (4), 121.0 (2,6)
**4e**	146.8	105.0	170.7	108.7	136.6	151.3	152.6	34.1 (Me)	13.9 (Me)	-	Ph: 136.7 (1), 129.6 (3,5), 128.1 (4), 121.5 (2,6)
**4f**	135.5	107.8	169.8	108.6	136.7	151.4	152.0	Ph: 159.9 (4), 129.4 (2,6), 126.2 (1), 114.4 (3,5); 55.3 (OMe), 52.4 (CH_2_)	-	-	Ph: 136.6 (1), 129.6 (3,5), 128.2 (4), 121.6 (2,6)
**4g**	146.9	105.5	170.7	108.8	136.6	151.3	152.4	Ph: 134.6 (1), 129.0 (3,5), 128.6 (4), 127.7 (2,6); 52.3 (CH_2_)	14.0 (Me)	-	Ph: 136.7 (1), 129.5 (3,5), 128.0 (4), 121.4 (2,6)
**5a**	138.1	117.8	192.1	117.8	138.1	145.5	145.5	Ph: 136.6 (1), 129.7 (3,5), 128.3 (4), 121.3 (2,6)	-	-	Ph: 136.6 (1), 129.7 (3,5), 128.3 (4), 121.3 (2,6)
**5b**	150.1	114.8	193.5	118.1	138.2	145.1	146.0	Ph: 136.4 (1), 129.56 (3,5), 128.0 (4), 121.2 (2,6)	15.6 (Me)	-	Ph: 136.6 (1), 129.6 (3,5), 128.2 (4), 121.1 (2,6)
**5d**	150.2	115.1	195.5	115.1	150.2	145.8	145.8	Ph: 136.5 (1), 129.6 (3,5), 127.9 (4), 121.2 (2,6)	15.9 (Me)	15.9 (Me)	Ph: 136.5 (1), 129.6 (3,5), 127.9 (4), 121.2 (2,6)
**5e**	148.8	113.5	193.5	117.7	138.1	145.3	147.3	34.2 (Me)	15.2 (Me)	-	Ph: 136.7 (1), 129.6 (3,5), 128.2 (4), 121.4 (2,6)
**5f**	136.9	117.0	192.2	117.5	138.1	145.5	146.2	Ph: 159.9 (4), 129.4 (2,6), 126.0 (1), 114.4 (3,5); 55.3 (OMe), 52.5 (CH_2_)	-	-	Ph: 136.5 (1), 129.6 (3,5), 128.3 (4), 121.5 (2,6)
**5g**	148.9	114.1	193.6	117.9	138.2	145.2	147.0	Ph: 134.4 (1), 129.1 (3,5), 128.7 (4), 127.8 (2,6); 52.4 (CH_2_)	15.4 (Me)	-	Ph: 136.6 (1), 129.6 (3,5), 128.1 (4), 121.3 (2,6)
**4x**	136.3	107.4	170.5	106.7	128.6	160.6	153.0	Ph: 136.5 (1), 129.6 (3,5), 128.0 (4), 121.8 (2,6)	-	-	-
**5x**	137.7	116.9	193.5	115.8	130.2	155.0	147.6	Ph: 136.3 (1), 129.6 (3,5), 128.2 (4), 121.8 (2,6)	-	-	-

**Table 4:** Selected ^13^C,^1^H spin coupling constants of **4a**-**b**, **4d**-**g**, **5a**-**b**, **5d**-**g**, **4x** and **5x** (Hz, solvents as listed in Table 2).

Comp	*J* of C-3	*J* of C-3a	*J* of C-4a	*J* of C-5	*J* of C-7a	*J* of C-8a	other couplings
**4a**	^1^*J* = 194.7	^2^*J*(H-3) = 9.9	^2^*J*(H-5) = 9.9	^1^*J* = 194.7	^3^*J*(H-5) = 5.2	^3^*J*(H-3) = 5.2	
**4b**	^2^*J*(3-Me) = 7.2	^3^*J*(3-Me) = 2.9	^2^*J*(H-5) = 10.0	^1^*J* = 194.4	^3^*J*(H-5) = 5.1		^1^*J*(3-Me) = 129.4
**4d**	*^2^J*(3-Me) = 7.1	^3^*J*(3-Me) = 2.7	^3^*J*(5-Me) = 2.7	^2^*J*(5-Me) = 7.1			^1^*J*(3-Me) = 129.3, ^1^*J*(5-Me) = 129.3
**4e**	^2^*J*(3-Me) = 7.1	^3^*J*(3-Me) = 2.6	^2^*J*(H-5) = 9.9	^1^*J* = 194.1	^3^*J*(H-5) = 5.2	^3^*J*(N-Me) = 2.1	^1^*J*(N-Me) = 141.7, ^1^*J*(3-Me) = 129.1
**4f**	^1^*J* = 194.1	^2^*J*(H-3) = 10.0	^2^*J*(H-5) = 9.9	^1^*J* = 194.6	^3^*J*(H-5) = 5.2	^3^*J*(H-3) ~ 5.2, ^3^*J*(N-CH_2_) = 2.8	^1^*J*(OMe) = 144.1, ^1^*J*(N-CH_2_) = 141.5, ^3^*J*(NCH_2_,Ph H-2,6) = 4.9, ^2^*J*(Ph C-1,NCH_2_) = 4.7, ^3^*J*(Ph C-2/6,NCH_2_) = 4.4
**4g**	^2^*J*(3-Me) = 7.1	^3^*J*(3-Me) = 2.8	^2^*J*(H-5) = 10.0	^1^*J* = 194.2	^3^*J*(H-5) = 5.2	^3^*J*(N-CH_2_) = 2.7	^1^*J*(N-CH_2_) = 141.0, ^1^*J*(3-Me) = 129.2, ^3^*J*(NCH_2_,Ph H-2,6) = 4.7
**5a**	^1^*J* = 195.8	^2^*J*(H-3) = 9.3	^2^*J*(H-5) = 9.3	^1^*J* = 195.8	^3^*J*(H-5) = 5.1	^3^*J*(H-3) = 5.1	
**5b**	^2^*J*(3-Me) = 7.1	^3^*J*(3-Me) = 2.6	^2^*J*(H-5) = 9.1	^1^*J* = 195.5	^3^*J*(H-5) = 5.0		^1^*J*(3-Me) = 129.7
**5d**	^2^*J*(3-Me) = 7.2	^3^*J*(3-Me) = 2.5	^3^*J*(5-Me) = 2.5	^2^*J*(5-Me) = 7.2			^1^*J*(3-Me) = 129.6, ^1^*J*(5-Me) = 129.6
**5e**	^2^*J*(3-Me) = 7.1	^3^*J*(3-Me) = 2.7	^2^*J*(H-5) = 9.1	^1^*J* = 195.3	^3^*J*(H-5) = 5.1	^3^*J*(N-Me) = 2.4	^1^*J*(N-Me) = 141.9, ^1^*J*(3-Me) = 129.4
**5f**	^1^*J* = 195.0	^2^*J*(H-3) = 9.4	^2^*J*(H-5) = 9.3	^1^*J* = 195.5	^3^*J*(H-5) = 5.1	^3^*J*(H-3) = 5.1, ^3^*J*(N-CH_2_) = 2.6	^1^*J*(OMe) = 144.1, ^1^*J*(N-CH_2_) = 141.4, ^3^*J*(NCH_2_,Ph H-2,6) = 4.6
**5g**	^2^*J*(3-Me) = 7.1	^3^*J*(3-Me) = 2.7	^2^*J*(H-5) = 9.2	^1^*J* = 195.3	^3^*J*(H-5) = 5.1	^3^*J*(N-CH_2_) = 2.8	^1^*J*(N-CH_2_) = 141.2, ^1^*J*(3-Me) = 129.5, ^3^*J*(NCH_2_, Ph H-2,6) = 4.4
**4x**	^1^*J* = 193.8	^2^*J*(H-3) = 10.2	^2^*J*(H-5) ~ 8.5	^1^*J* ~ 195.0	^3^*J*(H-5) =not resolved	^3^*J*(H-3) = 5.2	
**5x**	^1^*J* = 194.7	^2^*J*(H-3) = 9.5	^2^*J*(H-5) = 7.8	^1^*J* = 195.9	^3^*J*(H-5) = 8.5	^3^*J*(H-3) = 4.9	

**Table 5:** ^15^N-NMR chemical shifts of investigated compounds (δ in ppm, solvents as listed in Table 2).

Comp	N-1	N-2	N-6	N-7
**4a**	−186.8	−86.8	−86.8	−186.8
**4b**	−193.1	−93.6	−87.7	−187.1
**4d**	−193.4	−94.2	−94.2	−193.4
**4e**	−211.8	−91.7	−88.0	−187.5
**4f**	−191.8	−85.2	−87.5	−187.3
**4g**	−200.0	−91.3	−88.2	−187.4
**5a**	−187.6	−84.5	−84.5	−187.6
**5b**	−195.3	−92.1	−85.5	−188.0
**5d**	−196.2	−92.9	−92.9	−196.2
**5e**	−213.7	−89.7	−85.6	−188.2
**5f**	−192.4	−82.2	−84.9	−187.9
**5g**	−201.9	−89.5	−85.8	−188.2
**4x**	−186.4	−87.7	−179.2*	−179.2*
**5x**	−186.7	−84.3	−175.5*	−175.5*

* Not unambiguously classifiable.

#### 3.2.9. General procedure for the synthesis of **9a** and **9b**

According to a known procedure [24], to a solution of the corresponding carboxylic acid **7** (5 mmol) in absolute ethanol (30 mL), H_2_SO_4_ (2 mL) was added and the mixture was refluxed for 8 h. After the reaction mixture was concentrated *invacuo*, the residue was neutralized with a saturated solution of NaHCO_3_ and then extracted with dichloromethane (3 × 15 mL). Organic layers were combined and dried over sodium sulfate. The solvent was evaporated and the residue was purified by column chromatography (silica gel, mobile phase CH_2_Cl_2_:MeOH/9:1).

*Ethyl 5-chloro-1-phenyl-1*H*-pyrazole-4-carboxylate* (**9a**). Starting from **7a** (1.11 g, 5 mmol) 1.02 g (81%) of compound **9b** were obtained as colorless crystals; m.p. 57 ºC (lit. [46] m.p. 59–60 ºC); ^1^H-NMR (500 MHz, CDCl_3_): δ (ppm) 8.12 (s, 1H, H-3), 7.44–7.56 (m, 5H, Ph-H), 4.36 (q, 7.1 Hz, 2H, OCH_2_), 1.38 (t, 7.1 Hz, 3H, Me); ^13^C-NMR (125 MHz, CDCl_3_): δ (ppm) 161.5 (C=O, ^3^*J*(CO,OCH_2_) = 3.3 Hz), 142.3 (C-3, ^1^*J*(C-3,H-3) = 193.6 Hz), 137.4 (Ph C-1), 131.1 (C-5, ^3^*J*(C-5,H-3) = 5.4 Hz), 129.2 (Ph C-4), 129.1 (Ph C-3,5), 125.5 (Ph C-2,6), 112.3 (C-4, ^2^*J*(C-4,H-3) = 8.7 Hz), 60.6 (OCH_2_, ^1^*J* = 147.7 Hz; ^2^*J*(OCH_2_,Me) = 4.4 Hz), 14.3 (Me, ^1^*J* = 127.1 Hz; ^2^*J*(Me,OCH_2_) = 2.7 Hz); ^15^N-NMR (50 MHz, CDCl_3_): δ (ppm) −162.0 (N-1), −75.8 (N-2); MS *m/z* (%): 250/252 (M^+^, 34/11), 222 (37), 205 (100), 77 (89), 51 (61).

*Ethyl 5-chloro-3-methyl-1-phenyl-1*H*-pyrazole-4-carboxylate* (**9b**). Starting from **7b** (1.18 g, 5 mmol) 688 mg (52%) of compound **9b** were obtained as colorless crystals; m.p. 72–73 ºC (lit. [47] m.p. 74 ºC); ^1^H-NMR (500 MHz, CDCl_3_): δ (ppm) 7.41 (m, 2H, Ph H-2,6), 7.36 (m, 2H, Ph H-3,5), 7.30 (m, 1H, Ph H-4), 4.24 (q, 7.1 Hz, 2H, OCH_2_), 2.43 (s, 3H, 3-Me), 1.27 (t, 7.1 Hz, 3H, Me); ^13^C-NMR (125 MHz, CDCl_3_): δ (ppm) 161.9 (CO, ^3^*J*(CO,CH_2_) = 3.1 Hz), 151.7 (C-3, ^2^*J*(C-3,3-Me) = 7.0 Hz), 137.2 (Ph-C-1), 130.8 (C-5), 128.7 (Ph C-3,5), 128.5 (Ph C-4), 125.1 (Ph C-2,6), 109.8 (C-4, ^3^*J*(C-4,3-Me) = 2.7 Hz), 59.9 (OCH_2_, ^1^*J* = 147.6 Hz, ^2^*J*(OCH_2_,CH_2_) = 4.4 Hz), 14.4 (3-Me, ^1^*J* = 129.2 Hz), 13.9 (ester-Me, ^1^*J* = 127.0 Hz, ^2^*J*(CH_3_,CH_2_) = 2.6 Hz); ^15^N-NMR (50 MHz, CDCl_3_): δ (ppm) −168.2 (N-1), −77.6 (N-2); MS *m/z* (%): 264/266 (M^+^, 53/18), 219 (100), 155 (12), 77 (56), 51 (26).

## 4. Conclusions

Starting from appropriately substituted 2-pyrazolin-5-ones we have presented a widely applicable method for the preparation of substituted 1*H*-pyrano[2,3-*c*:6,5-*c*]dipyrazol-4(7*H*)-ones. Moreover, conversion of the latter into the corresponding thiones has been performed. Detailed NMR spectroscopic studies of the title compounds and their precursors were provided. 

## References

[1] Pinto M.M.M., Sousa M.E., Nascimento M.S.J. (2005). Xanthone Derivatives: New insights in biological activities. Curr. Med. Chem..

[2] El-Seedi H.R., El-Barbary M.A., El-Ghorab D.M.H., Bohlin L., Borg-Karlson A.-K., Goeransson U., Verpoorte R. (2010). Recent insights into the biosynthesis and biological activities of natural xanthones. Curr. Med. Chem..

[3] Na Y. (2009). Recent cancer drug development with xanthone structures. J. Pharm. Pharmacol..

[4] Suphavanich K., Maitarad P., Hannongbua S., Sudta P., Suksamrarn S., Tantirungrotechai Y., Limtrakul J. (2009). CoMFA and CoMSIA studies on a new series of xanthone derivatives against the oral human epidermoid carcinoma (KB) cancer cell line. Monatsh. Chem..

[5] Woo S., Jung J., Lee C., Kwon Y., Na Y. (2007). Synthesis of new xanthone analogues and their biological activity test – Cytotoxicity, topoisomerase II inhibition, and DNA cross-linking study. Bioorg. Med. Chem. Lett..

[6] Demirkiran O. (2007). Xanthones in Hypericum: synthesis and biological activities. Topics Heterocycl. Chem..

[7] Fotie J., Bohle D.S. (2006). Pharmacological and biological activities of xanthones. Anti-infective Agents Med. Chem..

[8] Kleemann A., Engel J., Kutscher B., Reichert D. (2001). Pharmaceutical Substances: Syntheses, Patents, Applications.

[9] Eller G.A., Wimmer V., Haring A.W., Holzer W. (2006). An Efficient Approach to Heterocyclic Analogues of Xanthone: A Short Synthesis of all possible Pyrido[5,6]pyrano[2,3-*c*]pyrazol-4(1*H*)-ones. Synthesis.

[10] Eller G.A., Haring A.W., Datterl B., Zwettler M., Holzer W. (2007). Tri- and Tetracyclic Heteroaromatic Systems: Synthesis of Novel Benzo-, Benzothieno- and Thieno-Fused pyrano[2,3-*c*]pyrazol-4(1*H*)-ones. Heterocycles.

[11] Eller G.A., Holzer W. (2007). A Convenient Approach to Heterocyclic Building Blocks: Synthesis of Novel Ring Systems Containing a [5,6]pyrano[2,3-*c*]pyrazol-4(1*H*)-one Moiety. Molecules.

[12] Eller G.A., Datterl B., Holzer W. (2007). Pyrazolo[4’,3’:5,6]pyrano[2,3-*b*]quinoxalin-4(1*H*)-one: Synthesis and Characterization of a Novel Tetracyclic Ring System. J. Heterocycl. Chem..

[13] Eller G.A., Wimmer V., Holzer W. (2007). Synthesis of Novel Polycyclic Ring Systems Containing two Pyrano[2,3-*c*]pyrazol-4(1*H*)-one Moieties. Khim. Geterotsikl. Soedin..

[14] Eller G.A., Habicht D., Holzer W. (2008). Synthesis of a Novel Pentacycle: 8-Methyl-10-phenylpyrazolo[4’,3’:5,6]pyrano[3,2-*c*][1,10]phenanthrolin-7(10*H*)-one. Khim. Geterotsikl. Soedin..

[15] Eller G.A., Zhang Q., Habicht D., Datterl B., Holzer W. (2009). Synthesis and NMR Data of Pyrazolo[4’,3’:5,6]pyrano[2,3-*b*]pyrazin-4(1*H*)-ones: Derivatives of a Novel Tricyclic Ring System. Acta Chim. Slov..

[16] Batezila G., Holzer W. (2010). (2-Chlorophenyl)-3-methylchromeno[2,3-*c*]pyrazol-4(1*H*)-one. Molbank.

[17] Jensen B.S. (1959). The Synthesis of 1-Phenyl-3-methyl-4-acyl-pyrazolones-5. Acta Chem. Scand..

[18] Williams A.C., Camp N. (2003). Product Class 4: Benzopyranones and Benzopyranthiones. Sci. Synth..

[19] Huemer V., Eller G.A., Holzer W. (2010). Heterocyclic analogs of xanthiones: 5,6-fused 3-methyl-1-phenylpyrano[2,3-*c*]pyrazol-4(1*H*)thiones – synthesis and NMR (^1^H, ^13^C, ^15^N) data. Magn. Reson. Chem..

[20] Huemer V., Holzer W. (2010). 1-Phenylpyrazolo[4’,3’:5,6]pyrano[2,3-*c*]pyridine-4(1*H*)thione. Molbank.

[21] Eller G.A., Holzer W. (2004). The 4-methoxybenzyl (PMB) function as a versatile protecting group in the synthesis of N-unsubstituted pyrazolones. Heterocycles.

[22] Jones G., Stanforth S.P. (1997). The Vilsmeier reaction of fully conjugated carbocycles and heterocycles. Org. React..

[23] Zhang X.-Li, Lu X.-H., Jiang W.-Q. (2008). Synthesis of 5-chloro-N-[[(fluorophenyl)amino]­thioxomethyl]-3-methyl-1-phenyl-1*H*-pyrazole-4-carboxamide derivatives and determination of their activity as agrochemical fungicides. Yingyong Huaxue.

[24] Baraldi P.G., Tabtizi M.A., Preti D., Bovero A., Fruttarolo F., Romagnoli R., Zaid N.A., Moorman A.R., Varani K., Borea P.A. (2005). New 2-Arylpyrazolo[4,3-c]quinoline Derivatives as Potent and Selective Human A_3_ Adenosine Receptor Antagonists. J. Med. Chem..

[25] Haider N., Heinisch G. (1986). Pyridazines. III. A Novel Approach to Pyrido[2,3-*d*]pyridazines by Annelation of the Pyridine Ring to the 1,2-Diazine System. Synthesis.

[26] Cherkasov R.A., Kutyrev G.A., Pudovik A.N. (1985). Organothiophosphorus reagents in organic synthesis. Tetrahedron.

[27] Pedersen B.S., Scheibye S., Nilsson N.H., Lawesson S.-O. (1978). Studies on organophosphorus compounds. XX. Syntheses of thioketones. Bull. Soc. Chim. Belg..

[28] Jesberger M., Davis T.P., Barner L. (2003). Applications of Lawesson’s reagent in organic and organometallic syntheses. Synthesis.

[29] Sarenko A.S., Kvitko I.Ya., Efros L.S. (1972). Heterocyclic analoges of xanthones. II. C-Acylation of 5-pyrazolinones and synthesis of chromono[3,2-*d*]pyrazoles. Khim. Geterotsikl. Soedin..

[30] Braun S., Kalinowski H.-O., Berger S. (1998). 150 and More Basic NMR Experiments.

[31] Bax A. (1984). Structure determination and spectral assignment by pulsed polarization transfer via long-range proton-carbon-13 couplings. J. Magn. Reson..

[32] Jippo T., Kamo O., Nagayama N. (1986). Determination of long-range proton-carbon 13 coupling constants with selective two-dimensional INEPT. J. Magn. Reson..

[33] Kalchhauser H., Robien W. (1985). CSEARCH: A Computer Program for Identification of Organic Compounds and Fully Automated Assignment of Carbon-13 Nuclear Magnetic Resonance Spectra. J. Chem. Inf. Comput. Sci..

[34] (2010). NMR Predict, version 4.7.

[35] (2008). ACD/C+H Predictors and DB, version 12.0.

[36] Huemer V., Eller G.A., Holzer W. (2010). Heterocyclic analogs of xanthiones: 5,6-fused 3-methyl-1-phenylpyrano[2,3-*c*]pyrazol-4(1*H*)thiones – synthesis and NMR (^1^H, ^13^C, ^15^N) data. Magn. Reson. Chem..

[37] Holzer W., Kautsch C., Laggner C., Claramunt R.M., Perez-Torralba M., Alkorta I., Elguero J. (2004). On the tautomerism of pyrazolones: the geminal ^2^*J*[pyrazole C-4,H-3(5)] spin coupling constant as a diagnostic tool. Tetrahedron.

[38] Begtrup M., Boyer G., Cabildo P., Cativiela C., Claramunt R.M., Elguero J., Ignacio Garcia J., Toiron C., Vesdo P. (1993). Carbon-13 NMR of pyrazoles. Magn. Reson. Chem..

[39] Sucrow W., Slopianka M. (1978). Enehydrazines, 19. Pyrazolium Betaines from 1,1-Dialkylhydrazines and Acetylenecarboxylic Esters. Chem. Ber..

[40] (2010). ACD Name, version 12.0.

[41] Eller G.A. (2006). Improving the quality of published chemical names with nomenclature software. Molecules.

[42] Koshelev Yu.N., Kvitko I.Ya., Efros L.S. (1972). Transfer of substituent effects in thieno[3,2-d]pyrazole. Zh. Org. Khim..

[43] Brack A. (1965). Condensed pyrazolopyridines. Liebigs Ann. Chem..

[44] Dickopp H. (1974). Sydnones. II. 5-Halopyrazolecarboxylic acids. Chem. Ber..

[45] Duquenois P., Amal H. (1942). The action of PCl_5_ and SOCl_2_ on antipyrine-4-carboxylic acid. Bull. Soc. Chim. Fr..

[46] Beck J.R., Gajewski R.P., Lynch M.P., Wright F.L. (1987). Nonaqueous Diazotiation of 5-Amino-1-aryl-1*H*-pyrazole-4- carboxylate Esters. J. Het. Chem..

[47] Rojahn C.A., Fahr K. (1923). Synthesis of pyrazole aldehydes. I. Liebigs Ann. Chem..

